# Sustained-Release Nanocapsules Enable Long-Lasting Stabilization of Li Anode for Practical Li-Metal Batteries

**DOI:** 10.1007/s40820-020-00514-1

**Published:** 2020-08-28

**Authors:** Qianqian Liu, Yifei Xu, Jianghao Wang, Bo Zhao, Zijian Li, Hao Bin Wu

**Affiliations:** School of Materials Science and Engineering, Zhejiang University, Hangzhou, 310027 People’s Republic of China

**Keywords:** Metal–organic frameworks, LiNO_3_, Nanocapsules, Lithium-metal anode, Lithium-metal batteries

## Abstract

A robust solid-electrolyte interphase (SEI) enabled by electrolyte additive is a promising approach to stabilize Li anode and improve Li cycling efficiency. However, the self-sacrificial nature of SEI forming additives limits their capability to stabilize Li anode for long-term cycling. Herein, we demonstrate nanocapsules made from metal–organic frameworks for sustained release of LiNO_3_ as surface passivation additive in commercial carbonate-based electrolyte. The nanocapsules can offer over 10 times more LiNO_3_ than the solubility of LiNO_3_. Continuous supply of LiNO_3_ by nanocapsules forms a nitride-rich SEI layer on Li anode and persistently remedies SEI during prolonged cycling. As a result, lifespan of thin Li anode in 50 μm, which experiences drastic volume change and repeated SEI formation during cycling, has been notably improved. By pairing with an industry-level thick LiCoO_2_ cathode, practical Li-metal full cell demonstrates a remarkable capacity retention of 90% after 240 cycles, in contrast to fast capacity drop after 60 cycles in LiNO_3_ saturated electrolyte.

**Electronic supplementary material:**

The online version of this article (10.1007/s40820-020-00514-1) contains supplementary material, which is available to authorized users.

## Highlights

- Nanocapsules made from metal–organic frameworks were designed for sustained release of additive (LiNO_3_) to passivate Li anode in commercial carbonate-based electrolyte.
- The nanocapsules with continuous supply of LiNO_3_ formed a nitride-rich solid electrolyte interphase layer on Li anode and persistently remedied the interphase during prolonged cycling.
- The practical Li-metal full cell delivered a prolonged lifespan with 90% capacity retention after 240 cycles which has been hardly achieved in commercial electrolyte.

**Electronic supplementary material:**

The online version of this article (10.1007/s40820-020-00514-1) contains supplementary material, which is available to authorized users.

## Graphical abstract

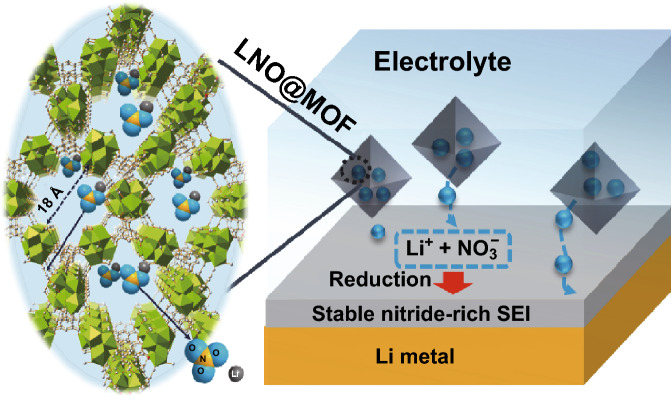

## Introduction

Li-metal batteries (LMBs) have attracted great attention in recent years due to the much improved energy density enabled by the use of Li-metal anode (LMA). However, Li dendrites growth and low Li cycling efficiency result in quick failure of the electrode and safety hazards [1–4]. Moreover, a prerequisite of high-energy-density LMBs is the successful adaptation of thin LMA with limited Li, which requires effective methods to improve the Columbic efficiency (CE) of LMA [3, 5, 6]. Solid-electrolyte interphase (SEI) layer is undoubtedly the key for durable LMA. A mechanically and chemically robust SEI layer with high ionic conductivity promotes homogeneous ion flux and uniform Li deposition/dissolution, thus minimizing dendrites formation and improving the CE of LMA [7–11]. Incorporating additives in electrolyte that are preferentially reduced on LMA (e.g., fluoroethylene carbonate (FEC) [12], lithium difluoro(oxalato)borate (LiDFOB) [13], and LiNO_3_ [14]) represents a feasible and efficient approach to manipulate the SEI [15–17].

In particular, LiNO_3_ has been demonstrated as an effective additive or co-salt in ether-based electrolytes for Li–S and Li–O_2_ batteries, forming stable SEI on LMA enriched with inorganic compounds of LiN_*x*_O_*y*_ and Li_3_N [18–20]. Such highly ionic conductive SEI facilitates Li^+^ transport and regulates the deposited Li into granular structure, which enhances Li cycling efficiency and prohibits dendrite-induced short circuits. Nevertheless, adaptation of LiNO_3_ in carbonate-based electrolytes, which are mature and compatible with high-voltage cathodes (e.g., LiCoO_2_ and LiNi_*x*_Co_*y*_Mn_*z*_O_2_), has been limited by its extremely low solubility in carbonate solvents (about 0.8 mg mL^−1^) [21]. CuF_2_ has been found to increase the solubility of LiNO_3_ to 1.0 wt % (10 mg mL^−1^) in carbonate electrolyte by forming Cu^2+^-NO_3_^−^ complex [22], yet the possible reduction of Cu^2+^ might compromise the stability of this system. Alternatively, solid LiNO_3_ has been incorporated in LMBs by pre-impregnating on porous separator [23], intercalating between bilayer separators [24], or encapsulating in polymer gel [21, 25] to overcome the solubility limit of LiNO_3_, however, at the cost of extra weight gain and possibly blocked ionic transport. To this end, efficient method to continuously stabilize LMAs with high-ionic conductivity nitride-rich SEI in carbonate-based electrolyte for practical LMBs has not been achieved.

Inspired by drug delivery systems for disease treatment [26, 27], we demonstrate sustained-release nanocapsules made from nanoparticles of metal–organic frameworks (MOFs) to continuously stabilize LMA with LiNO_3_ additive in carbonate-based electrolyte. While MOFs have been used to immobilize ionic species as electrolytes for battery applications [28–31], there is no attempt to manipulate the SEI-forming components in commercial electrolytes with the assistance of MOFs. In this study, MOF nanoparticles can uptake a substantial amount of LiNO_3_ (denoted as LNO@MOF) and well disperse in liquid electrolyte. More than ten times of the solubility limit of LiNO_3_ can be introduced into electrolyte. During battery operation, LiNO_3_ is continuously consumed to remedy the SEI layer and replenished by the nanocapsules. Here we show that thin LMA of 50 μm can be effectively stabilized in commercial carbonate-based electrolyte containing LNO@MOF nanocapsules. When coupled with a thick LiCoO_2_ cathode (21 mg cm^−2^), the as-assembled practical LMB full cell demonstrates outstanding cycle stability with a capacity retention of 90% after 240 cycles.

## Experimental Section

### Synthesis of MOF-808 Nanoparticles

MOF-808 nanoparticles were prepared according to a previous report [32]. Briefly, ZrOCl_2_·8H_2_O (0.97 mg, 3.0 mmol) and 1,3,5-benzenetricarboxylate (H_3_BTC) (0.21 mg, 1 mmol) were dissolved in *N*,*N*-dimethylformamide (DMF)/formic acid (30 mL/30 mL) and loaded into a 100-mL Teflon-lined autoclave and heated at 130 °C for two days. After cooling to room temperature, the MOF-808 power was collected by filtration and washed by DMF for three times. Afterward, the sample was immersed in methanol for solvent exchange for 3 days, during each time methanol was replaced three times per day. Finally, MOF-808 was vacuum-dried at room temperature and then at 150 °C for 10 h to yield MOF-808 nanoparticles.

### Preparation of LNO@MOF Nanocapsules

The MOF-808 nanoparticles were immersed into 2 mol L^−1^ LiNO_3_ methanol solution and stirred for 24 h to enable full impregnation of LiNO_3_ into the pores of MOF-808. Then, the composites were washed by methanol for three times to remove residual LiNO_3_ outside MOF-808 particles. Finally, LNO@MOF was vacuum-dried at 100 °C for 10 h to remove methanol solvent.

### Preparation of LNO@MOF Electrolyte

In an argon-filled glovebox, LNO@MOF nanocapsules were added into blank electrolyte in which 1 M LiPF_6_ was dissolved in ethylene carbonate (EC)/diethyl carbonate (DEC) (1/1, by volume) to reach certain concentrations (25, 50 and 100 mg mL^−1^). The obtained electrolyte was stirred for 2 h to ensure uniform dispersion. LNO@MOF electrolyte containing 50 mg mL^−1^ of LNO@MOF is used unless specified.

### Materials Characterizations

The structure of MOF-808 and LNO@MOF was characterized by powder X-ray diffractometer (XRD, Lab XRD-6000). Thermogravimetric analysis (TGA, SDT Q600) was performed in the temperature range in N_2_ atmosphere between 30 and 800  °C by a ramping rate of 5 °C min^−1^. The N_2_ adsorption–desorption isotherms of MOF-808 and LNO@MOF were measured by Autosorb-IQ-MP (Quantachrome Instruments) at 77 K. The Li/Zr molar ratio of LNO@MOF was measured using the inductive coupled plasma optical emission spectrometer (ICP-OES, Varian 730-ES). The sample was prepared by dissolving LNO@MOF into nitric acid (HNO_3_) and diluted to a certain volume. The UV–Vis absorption spectra of the LNO@MOF in electrolyte were recorded by MAPADA P4 system at 25 °C. Specifically, the LNO@MOF or MOF electrolyte was first centrifuged and the supernatant electrolyte was acquired. The sample was prepared by dissolving a certain amount of supernatant electrolyte (10 μL) into deionized water (10 mL) and then transferred into 10-mm quartz cells. Afterward, the supernatant electrolyte was removed and refreshed with blank electrolyte. The refreshed electrolyte was uniformly dispersed by ultrasonic and rested for 2 h to ensure the release of LiNO_3_ from nanocapsules. Again, the LNO@MOF electrolyte was centrifuged, and the supernatant electrolyte was acquired for UV measurement. MOF-808 electrolyte (MOF-808 dispersed in electrolyte) was used as reference sample for LNO@MOF electrolyte and blank electrolyte for LiNO_3_ saturated electrolyte. The data were measured in the range of 300–190 nm. Scanning electron microscopy (SEM) images and X-ray energy-dispersive spectroscopy (EDS) were performed using Phenom LE. X-ray photoelectron spectroscopy (XPS) analysis was performed on Thermo Fisher Scientific Escalab 250Xi. For SEM and XPS analyses, the electrode disassembled in the argon filled glovebox was washed by dimethyl carbonate (DMC) for three time and vacuum-dried. A sealed container was used before transferring for further postmortem analysis. Electrolyte ionic conductivity was measured by electrochemical impedance spectroscopy with a two-electrode stainless steel cell with the cell constant of 0.637 cm^−1^ at 25 °C. Viscosity of electrolytes was measured using HAAKE Rotational Rheometer (RS6000) with the parallel plate at a shear rate of 100 s^−1^ at 25 °C.

### Electrochemical Measurements

Li|Cu, Li|Li and Li|LiCoO_2_ cells were prepared using CR2032 coin cells with 40 μL electrolyte in each cell and polypropylene membranes as the separator. The coin cells were monitored by a battery testing system (Neware, CT-4008-5V10 mA) at 27 °C. The average Coulombic efficiency of Li|Cu coin cell was calculated based on a method developed by Zhang et al. [33]. Specifically, 5 mAh cm^−2^ of Li was first plated on the Cu substrate and stripped to 1 V before depositing the Li reservoir (*Q*_T_ = 5 mAh cm^−2^) at 0.5 mA cm^−2^, then cycling (*Q*_C_ = 0.5 mAh cm^−2^) for 20 cycles (*n* = 20), and finally dissolving all the remaining Li (*Q*_S_) to 1 V. The average CE over n cycles is calculated as follows:

1 $$ {\text{CE}}_{\text{ave}} = \, \left( {Q_{\text{S}} + \, nQ_{\text{C}} } \right)/\left( {Q_{\text{T}} + \, nQ_{\text{C}} } \right) $$

Electrochemical impedance spectroscopy (EIS) test was measured on a Bio-Logic SAS (MPG2) with a frequency range of 20 kHz–10 mHz and an applied voltage of 10 mV at 27 °C. LiCoO_2_ cathode was prepared by mixing active material, super P and poly(vinylidene fluoride) (PVDF) with weight ratios of 96:2:2 in *N*-methyl-2-pyrrolidone (NMP) and blade coated on Al foil and vacuum-dried at 80 °C. Areal loading of LiCoO_2_ cathode was around 21 mg cm^−2^. The cycled LCO|Li full cell was dissembled, and the Li anode was reassembled with stainless steel (SS) in blank electrolyte to measure the remaining capacity of the cycled lithium anode. The Li|SS cell was charged to 1.0 V at a current density of 0.2 mA cm^−2^.

## Results and Discussion

### Synthesis and Characterizations of LNO@MOF Nanocapsules

**Fig. 1:**
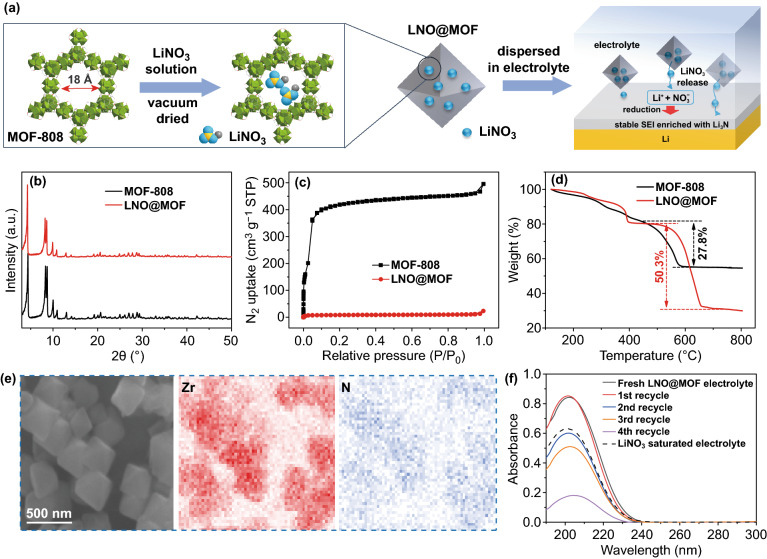
**a** Schematic illustration of LiNO_3_ encapsulated into MOF-808 and its sustained release in electrolyte. **b** XRD patterns, **c** N_2_ adsorption–desorption isotherms, and **d** TGA of MOF-808 and LNO@MOF. **e** SEM image and elemental mappings of LNO@MOF. **f** UV absorption spectra of NO_3_^−^ in electrolyte released from LNO@MOF

Schematic illustration of the synthesis and working principle of the LNO@MOF nanocapsules is shown in Fig. 1a. MOF-808 was chosen as the host of nanocapsules due to its high surface area, large pore volume, high (electro-)chemical stability in organic electrolyte, easy synthesis and relatively low cost. The large adamantane cage in MOF-808 with an internal diameter of 18.4 Å and a pore window of 14 Å enables efficient encapsulation and diffusion of LiNO_3_ [34]. The LNO@MOF nanocapsules were prepared by impregnating MOF-808 with LiNO_3_ methanol solution, followed by centrifugation and vacuum drying. The as-synthesized LNO@MOF nanocapsules can be easily dispersed in carbonate-based electrolyte, which release LiNO_3_ into electrolyte until saturation. Consumption of LiNO_3_ to form SEI on fresh Li would be rapidly compensated by the LNO@MOF nanocapsules, which keep the electrolyte in a LiNO_3_-saturated quasi-equilibrium state.

The as-prepared LNO@MOF was characterized by XRD as shown in Fig. 1b. The XRD pattern of the LNO@MOF is closely matched with that of MOF-808, confirming that the crystal structure of MOF-808 remains intact after loading LiNO_3_. The absence of diffraction peaks from LiNO_3_ confirms its encapsulation in the micropores of MOF-808 without long-range order. As shown by the N_2_ adsorption–desorption isotherms of MOF-808 (Figs. 1c and S1), the specific surface area and pore volume of LNO@MOF are substantially reduced from 765.4 m^2^ g^−1^ and 0.638 cm^3^ g^−1^ (MOF-808) to 19.6 m^2^ g^−1^ and 0.02 cm^3^ g^−1^, respectively, after incorporation of LiNO_3_. The reduced porosity verifies the hypothesis that LiNO_3_ is mainly encapsulated into the pores of MOF-808 rather than precipitates alongside the MOF particles. Thermogravimetric analysis (TGA) of LNO@MOF further verifies the successful incorporation of LiNO_3_ as shown in Fig. 1d. A much higher weight loss of about 50 wt% between 400 and 800 °C is observed for LNO@MOF compared to 28 wt% for pristine MOF-808, which could be attributed to the decomposition of LiNO_3_ into Li_2_O. Scanning electron microscopy (SEM) shows similar morphology of MOF-808 and LNO@MOF consisting of octahedral particles of ~ 500 nm (Fig. S2). Uniform distribution of N and Zr elements in LNO@MOF is revealed by X-ray energy-dispersive spectroscopy (EDS) elemental mapping (Fig. 1e). The loading amount of LiNO_3_ in LNO@MOF is estimated at ~ 21 wt% based on inductively coupled plasma-optical emission spectrometry (ICP-OES) and EDS analysis (Table S1).

LNO@MOF nanocapsules can be easily dispersed in commercial carbonate-based electrolyte (1 M LiPF_6_ in ethylene carbonate (EC)/diethyl carbonate (DEC) of 1/1 by volume) with a concentration of 50 mg mL^−1^ to form a colloidal electrolyte (denoted as LNO@MOF electrolyte, Fig. S3a), corresponding to a NO_3_^−^ concentration of 11 mg mL^−1^ (0.16 mol L^−1^). LNO@MOF electrolyte exhibits slightly increased viscosity and reduced ionic conductivity compared with pristine electrolyte (Table S2), and maintains good wettability with separators (Fig. S3b). The sustained release of LiNO_3_ from nanocapsules into electrolyte was monitored by ultraviolet light absorption (Fig. 1f) according to the strong absorption peak of NO_3_^−^ ion at around 205 nm [35, 36]. Surprisingly, the absorbance of NO_3_^−^ in LNO@MOF electrolyte is notably higher than that of LiNO_3_ saturated electrolyte, implying a supersaturated NO_3_^−^ solution. A possible explanation is the absorption of PF_6_^−^ by MOF-808 [28], which reduces the PF_6_^−^ concentration and in turn increases NO_3_^−^ solubility [21]. After refreshing electrolyte, LiNO_3_ concentration remains a high value until the third recycled electrolyte, revealing that nanocapsules help to maintain the LiNO_3_-saturated state of electrolyte, ensuring its long-term effect as a consumed additive during cycling.

### Electrochemical Performance of LMA in LNO@MOF Electrolyte

**Fig. 2:**
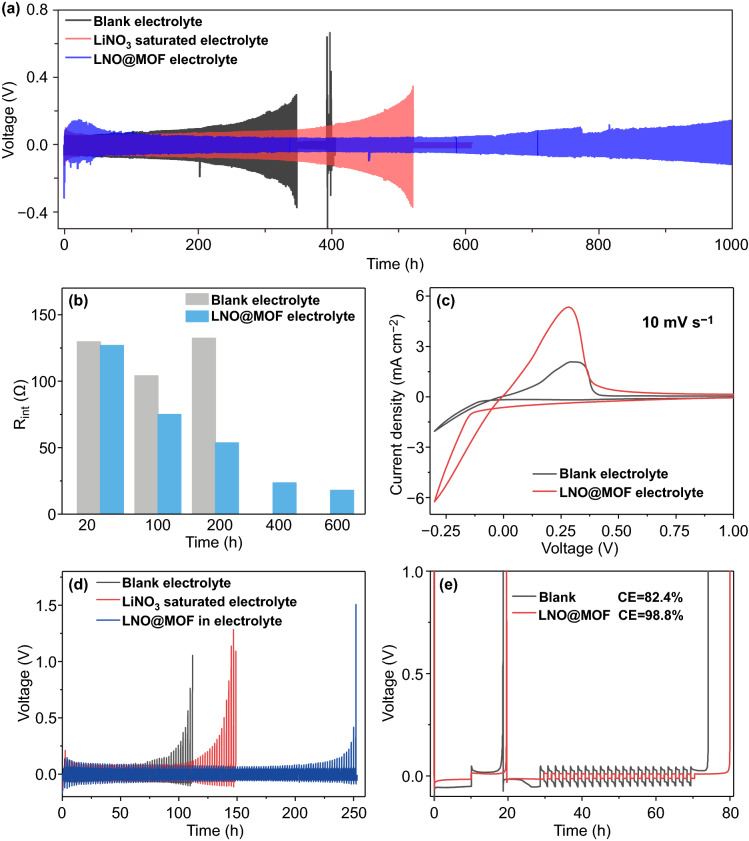
**a** Voltage profiles of Li|Li symmetric cells at 1.0 mA cm^−2^ to a capacity depth of 1.0 mAh cm^−2^ and **b** *R*_int_ of Li|Li symmetric cells during cycling obtained from Nyquist plot. **c** CVs of Cu|Li cells with blank and LNO@MOF electrolytes at a scan rate of 10 mV s^−1^. **d** Voltage profiles of asymmetric 50 μm-Li|Li cells at 1.0 mA cm^−2^ with a cycled capacity of 1.0 mAh cm^−2^. **e** Voltage–time profiles to calculate the average Coulombic efficiency of LMA at 0.5 mA cm^−2^

The interfacial stability of LMA in LNO@MOF electrolyte is studied by Li|Li symmetric cells. As shown in Fig. 2a, the cycling stability of the cell with LiNO_3_ saturated electrolyte is improved compared with that of blank electrolyte, however still fails after 530 h. Thus, LiNO_3_ indeed enables a more stable interphase on LMA, yet the interphase would fail upon prolonged cycling. When using LNO@MOF electrolyte, the cell can be cycled for more than 1000 h with a low overpotential below 100 mV and no sign of short circuit. The extended lifespan of the LMA in LNO@MOF electrolyte could be attributed to the continuous release of LiNO_3_, which immediately repairs the damaged SEI during cycling. The charge transport characteristic of interfacial layer formed on lithium anode is studied by EIS as shown in Figs. 2b and S3. The interfacial resistance (*R*_int_) represented by the semicircle at high-frequency region in Nyquist plots decreases during cycling for the Li|Li cell using LNO@MOF electrolyte, suggesting that the SEI is gradually stabilized and enriched with high ion-conductive species. This notably differs from the *R*_int_ of the cell with blank electrolyte, which decreases after the first 50 cycles and then increases afterward due to unstable SEI formation. The reaction kinetics of lithium plating/stripping was further examined by cyclic voltammetry (CV) tests of Cu|Li cells. As shown in Fig. 2c, the current response of initial Li plating/stripping is significantly enhanced in LNO@MOF electrolyte, corresponding to lower Li^+^ transfer barriers through SEI and fast reaction kinetics. This signifies the contribution of interface layer formed by LiNO_3_ decomposition that improves electrochemical kinetics of lithium deposition/dissolution.

Asymmetric cells were assembled using limited Li (50 μm Li corresponds to about 10 mAh cm^−2^) as the working electrode to evaluated the durability of practical LMA. The cutoff voltage of 1.0 V was set as the complete consumption of active Li in the 50 μm Li foil. Figure 2d shows that the cell using LNO@MOF electrolyte exhibits an extended cycle life of 250 h, which is much longer than that of LiNO_3_ saturated electrolyte (150 h) and blank electrolyte (112 h). Note that the concentration of LNO@MOF would be directly related to the maximal amount of released LiNO_3_, and a lower LNO@MOF concentration of 25 mg mL^−1^ leads to inferior cycling performance of the Li|Li cell. However, further increasing the concentration of LNO@MOF to 100 mg mL^−1^ leads to increased cell polarization (Fig. S5a), possibly due to the aggregation of nanocapsules that impedes the ionic transport. Our previous studies indicate that MOFs with abundant open-metal sites (OMSs) to immobilize anions would increase the Li^+^ ion transference number of liquid electrolyte and improve the stability of LMA [37, 38]. However, due to the few OMSs in low-temperature-treated MOF-808 and its relative low content, pristine MOF-808 in electrolyte only marginally improves the cycle life of LMA (Fig. S5b). The improvement of LMA in LNO@MOF electrolyte would be mainly attributed to the released LiNO_3_ from nanocapsules.

The average Coulombic efficiency (CE) of Li stripping/plating was determined by Cu|Li cells. Figure 2e shows that the average CE of the cell with LNO@MOF is 98.8%, which is much higher than that of blank electrolyte (82.4%). The voltage polarization is also reduced in LNO@MOF electrolyte, which is in line with the observation in EIS measurement. The CE of Cu|Li cells during cycling in Fig. S6 also shows that the high CE with LNO@MOF electrolyte could be maintained for 100 cycles without obvious decay. The results confirm that the sustained release of LiNO_3_ from nanocapsules guarantees high lithium cycling efficiency with small voltage hysteresis.

### Li Deposition Morphologies and Characterization of SEI

**Fig. 3:**
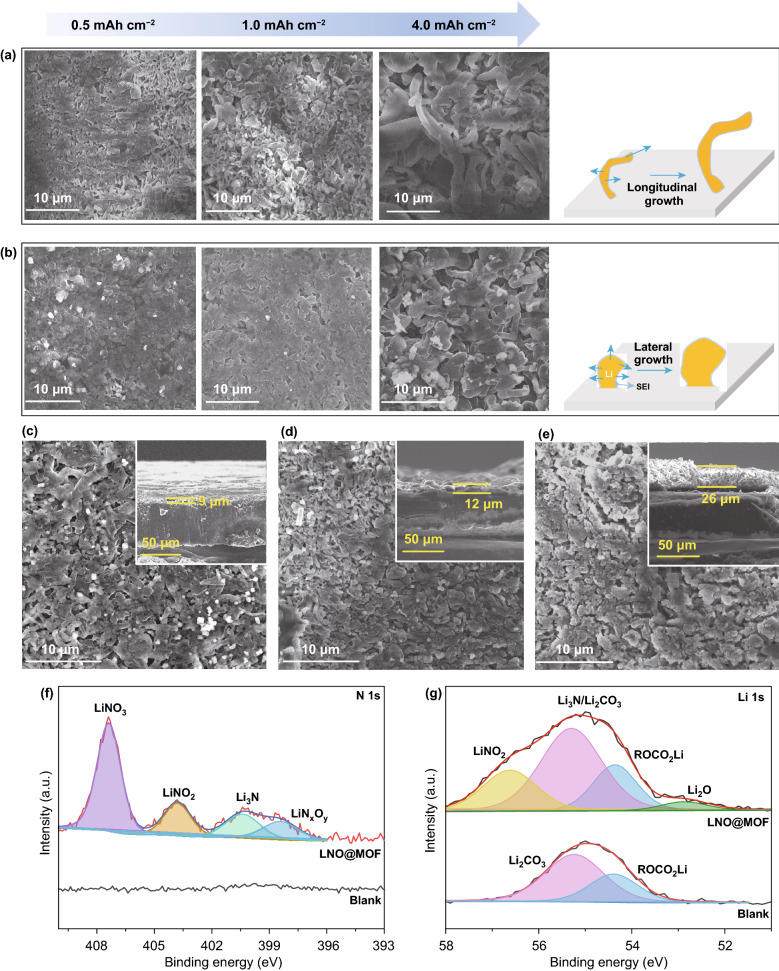
SEM images of LMA after deposition of 0.5, 1 and 4.0 mAh cm^−2^ at 1 mA cm^−2^ with **a** blank electrolyte and **b** LNO@MOF electrolyte. The surface and cross section images of the cycled LMA in LNO@MOF electrolyte after **c** 10 cycles, **d** 50 cycles, and **e** 100 cycles at 1 mA cm^−2^ and a capacity 1.0 mAh cm^−2^ (inset is the cross section view). XPS spectra of **f** N 1*s*, **g** Li 1*s* of the LMA with blank and LNO@MOF electrolyte after 10 cycles

The morphology of Li deposited on limited Li (50 μm) during cycling was examined by SEM. Figure 3a, b shows the morphology evolution of deposited Li with increased capacity at 1 mA cm^−2^. With blank electrolyte (Fig. 3a), Li grows into dendritic structures along longitudinal with increased capacity, which easily cause excessive SEI formation, isolated Li and short circuit. In contrast, with the LNO@MOF electrolyte (Fig. 3b), Li prefers to grow laterally into granular structures with good uniformity instead of dendritic or whisker growth. As the cycle number increases, the morphology of the deposited Li could be generally maintained even after 100 cycles as show in Fig. 3c–e. In contrast, the deposited Li presents a porous and loose structure with random inactive Li dendrites during cycling in blank electrolyte (Fig. S7). The cross section views (inset of Fig. 3c–e) present that the top layer containing deposited Li and passivation layer is relatively dense and smooth. The thickness increases slowly during cycling, indicating less severe Li pulverization during repeated plating and stripping. The intact active Li on the Li foil remains about 60% of the initial capacity of pristine Li foil after 100 cycles. The morphology of deposited Li during cycling signifies the LiNO_3_-induced stable SEI in reducing Li dendrite growth and “dead Li” for practical LMA.

XPS analysis and EDS were carried out to study the chemical composition of SEI layer on Li surface (Figs. S8 and S9), consisting of various inorganic and organic moieties. N 1*s* XPS spectrum in Fig. 3f demonstrates that the main decomposition products of LiNO_3_ on Li are Li_3_N, LiN_*x*_O_*y*_ and LiNO_2_. Moreover, SEI is enriched with various inorganic Li compounds, especially with abundant N-containing species as revealed by Li 1*s* XPS spectrum in Fig. 3g. This inorganic-rich SEI facilitates the de-solvation process and ion diffusion through SEI, thus ensuring a stable and efficient lithium plating/stripping during cycling [39]. In addition, such nitride-enriched SEI is rather dense to prevent the decomposition of LiPF_6_ even after prolonged cycling, as revealed by the XPS spectra and EDS (Figs. S8 and S9).

### Electrochemical Performance of Practical LMB

**Fig. 4:**
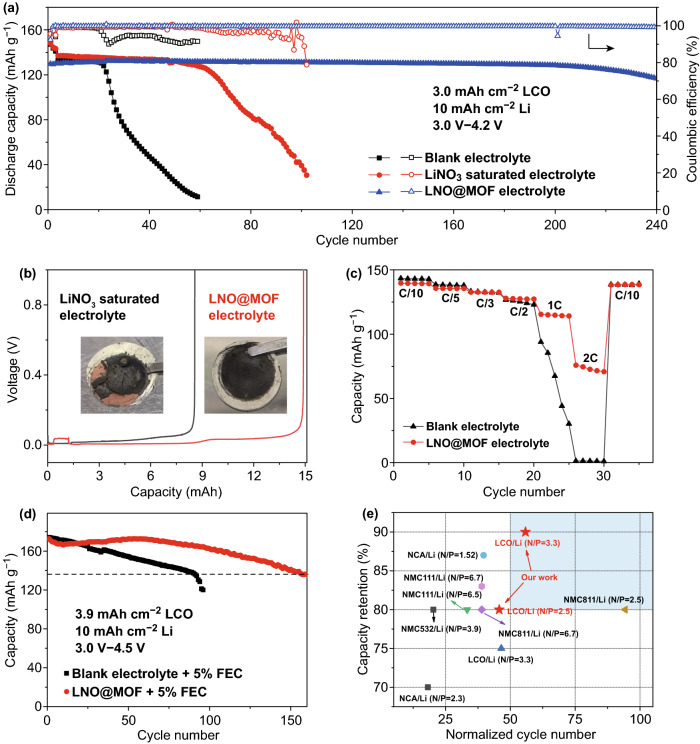
**a** Long-term cycling performance of a practical LCO|Li full cell with a N/P ratio of 3.3, cycled at 0.2 C charge/0.5 C discharge. **b** Quantification of remaining active Li after cycling. Inset: optical images of the Li anode disassembled from the cycled LCO|Li cell in (**a**). **c** Rate performance of LCO|Li full cell in different electrolytes. **d** Long-term cycling performance of LCO|Li full cell with a N/P ratio of 2.5, cycled at 0.2 C charge/0.5 C discharge with 5% FEC additive. **e** Summary of capacity retention of practical high-voltage LMBs shown in Table S2. The normalized cycle number refers to the total cycle number divided by the equivalents of total active Li with respect to cathode (i.e., N/P ratio plus one). The shadow region indicates a capacity retention higher than 80% after 200 cycles with a N/P ratio below 3

To demonstrate the implementation of LNO@MOF electrolyte in high-energy LMBs, we fabricate full cells using LiCoO_2_ (LCO) as cathode and thin LMA. Notably, a low negative-to-positive-capacity (N/P) ratio is imperative for high-energy density LMB [3, 6]. Hence, a commercial-level LCO cathode of around 3.0 mAh cm^−2^, a limited Li (50 μm, 10 mAh cm^−2^) with a N/P ratio of around 3.3 and lean electrolyte of 13 μL mAh^−1^ were employed. Figure 4a shows that LCO|Li full cells with blank and LiNO_3_ saturated electrolyte could operate stably for 21 and 62 cycles, respectively, followed by rapid decline in capacity and CE that is likely due to the fast consumption of the limited Li [7]. Notably, the full cell with LNO@MOF electrolyte demonstrates an extraordinary cycle stability with a capacity retention of 90% after 240 cycles. Voltage profiles (Fig. S10) also present low polarization for the cell with LNO@MOF electrolyte in comparison with control cells with blank and LiNO_3_ saturated electrolytes.

To quantify the exact Li loss during cycling, cycled Li anode was reassembled against a stainless steel (SS) electrode to measure the remaining active Li. Figure 4b shows 8.5 mAh of Li remains on the cycled LMA in LiNO_3_ saturated electrolyte, which is consistent with original Li from the extra area of anode compared to cathode (0.87 cm^−2^ corresponds to 8.7 mAh). Therefore, there is actually no active Li remains in the cycled area. In contrast, 14.9 mAh of Li remains in LNO@MOF electrolyte, which corresponds to 5.5 mAh cm^−2^ (~ 55% of the initial capacity) of active Li remains in the cycled area. Optical image of the cycled LMA (inset in Fig. 4b) confirms the less corroded appearance in LNO@MOF electrolyte compared with pulverized black powders detached from current collector in LiNO_3_ saturated electrolyte. The SEM morphology of cycled lithium in Fig. S11 shows similar results. For LNO@MOF electrolyte, the cycled Li surface is smoother and more compact, in contrast to an uneven surface with cracks and lithium dendrites in LiNO_3_ saturated electrolyte. Cross section image of the cycled Li foil shows around 30 μm active Li (60% of initial capacity, Fig. S11) remains after 240 cycles in LNO@MOF electrolyte, while no active Li remains in LiNO_3_ saturated electrolyte, which agrees well with the residual lithium from the reassemble Li|SS cell (Fig. 4b). Moreover, the passivation layer (16 μm) is much thinner than that (40 μm) of the cycled Li in LiNO_3_ saturated electrolyte. LCO|Li cell with thick Li anode (400 μm) and thin LCO cathode (1.5 mg cm^−2^) was also tested (Fig. S12). Cycling stability of the cell with LNO@MOF electrolyte outperforms the cell with blank electrolyte, indicating reduced electrolyte consumption and Li pulverization during cycling due to the stable SEI formed in LNO@MOF electrolyte.

The rate capability of LCO|Li cells with LNO@MOF electrolyte was evaluated. As shown in Fig. 4c, for the cell with blank electrolyte, a rapid capacity decline occurs from 1 C and little capacity remains at 2 C. In comparison, the cell with LNO@MOF performs better capacity at high charge rates with the stable specific capacities of 128, 116 and 75 mAh g^−1^ at 0.5, 1 and 2 C, respectively, presenting rapid electrochemical kinetics at high rates for practical LMBs. The LCO|Li full cell in LNO@MOF electrolyte at higher cutoff voltage (4.5 V) was also studied. As shown in Fig. 4d, LCO|Li full cell with blank electrolyte displays a sharp decrease in capacity with only 69% capacity retention at the 100^th^ cycle. In contrast, with LNO@MOF electrolyte, the cycling performance greatly improves, retaining 80% of the capacity after 160 cycles. Voltage profiles shown in Fig. S13 reveal gradually increased polarization possibly related to cathode degradation at high potential, to which the inferior cycling stability could be attributed.

Capacity retentions of practical LMBs with high-voltage cathodes in recent studies are summarized in Fig. 4e and Table S3. Considering that durability of practical LMBs is strongly correlated with available active Li and different N/P ratios in previous studies prevent direct comparison of the cycle life, a normalized cycle number is adopted here (see caption of Fig. 4 for details). The cycling performance of LCO|Li cells with LNO@MOF electrolyte outperforms most of the reported results. In particular, the LCO|Li cell cycling between 3.0 and 4.2 V is in the long-cycle-life region (above 80% capacity retention for 200 cycles with a low N/P ratio below 3), where very few works have been achieved. The LNO@MOF nanocapsules in this work provide a promising solution to extend the cycle life of practical LMBs.

## Conclusions

In summary, we have demonstrated LNO@MOF nanocapsules by absorbing LiNO_3_ into pores of MOF-808 nanoparticles to stabilize LMA. The nanocapsules provide continuous release of LiNO_3_ into electrolyte and overcome the solubility limitation of LiNO_3_ in carbonate-based electrolyte. The dissolved LiNO_3_ preferentially reduced on LMA, forming robust and highly ionic conductive SEI that guarantees uniform Li deposition. As a result, the cycle life of thin Li anode in 50 μm increases from 112 to 250 h and a higher average Li cycling efficiency of 98.8% is obtained. Outstanding cycling performance is achieved in LCO|Li full cell with a low N/P ratio of 3.3, showing a high capacity retention of 90% after 240 cycles. This work demonstrates an effective strategy to utilize SEI-forming additives with low solubility for practical LMB.

## Electronic supplementary material

Below is the link to the electronic supplementary material.

Supplementary material 1 (PDF 1181 kb)
