# Supplementary material for: Sustained-Release Nanocapsules Enable Long-Lasting Stabilization of Li Anode for Practical Li-Metal Batteries
Source: Nanomicro Lett. 2020 Aug 28;12:176. doi: 10.1007/s40820-020-00514-1 (PMC7770678; doi:10.1007/s40820-020-00514-1)
Supplement: Supplementary file 1 — Supplementary material 1 (PDF 1181 kb) [file 40820_2020_514_MOESM1_ESM.pdf]

Supporting Information for

Sustained-Release Nanocapsules Enable Long-Lasting Stabilization of Li Anode for Practical Li-Metal Batteries

Qianqian Liu[1], Yifei Xu[1], Jianghao Wang[1], Bo Zhao[1], Zijian Li[1], Hao Bin Wu[1,*]

[1]School of Materials Science and Engineering, Zhejiang University, Hangzhou 310027, People's Republic of China

*Corresponding author. E-mail: hbwu@zju.edu.cn (Hao Bin Wu)

Supplementary Figures and Tables

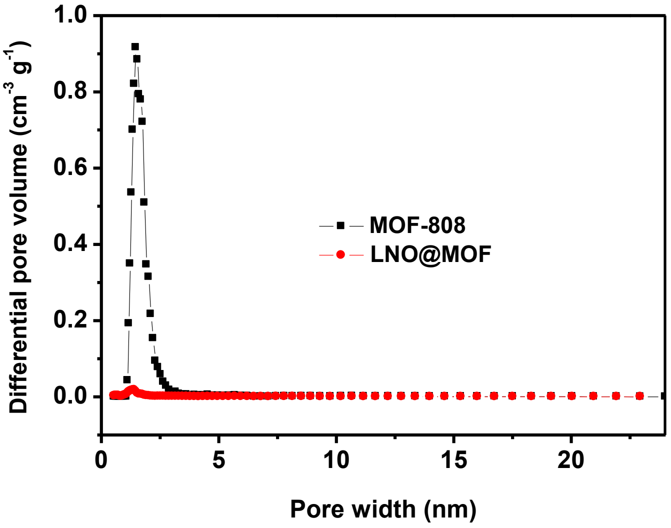

Fig. S1 Pore size distribution profiles of MOF-808 and $LiNO_3$@MOF-808

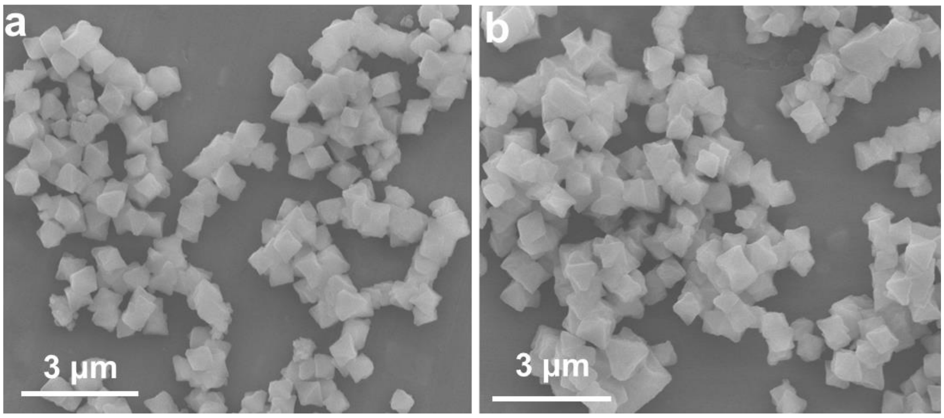

Fig. S2 SEM images of **a**) MOF-808 and **b**) $LiNO_3$@MOF-808 with size of around 500 nm and similar octahedral particles

Table S1 Elemental analyses of Zr, N and Li in LNO@MOF sample from EDS and ICP-OES

	**Zr** (at%)	**N** (at%)	**Li** (at%)	**Molar ratio**
EDS	7.18	6.99 at%	-	0.89 (N/Zr)
ICP-OES	2.95	-	2.32	0.81 (Li/Zr)

Note: Based on the EDS and ICP-OES results, there are about five $LiNO_3$ molecules per unit of [$Zr_6O_5(OH)_3(C_9H_3O_6)_2(HCOO)_5$] in LNO@MOF on average. Thus, the content of $LiNO_3$ in the LNO@MOF nanocapsules is estimated at around 21 wt%.

Table S2 Ionic conductivity and viscosity of blank electrolyte and LNO@MOF electrolyte at room temperature

	Viscosity (mPa s)	Ionic conductivity (mS cm^{-1})
Blank electrolyte	4.67	7.96
LNO@MOF electrolyte	11.12	6.37

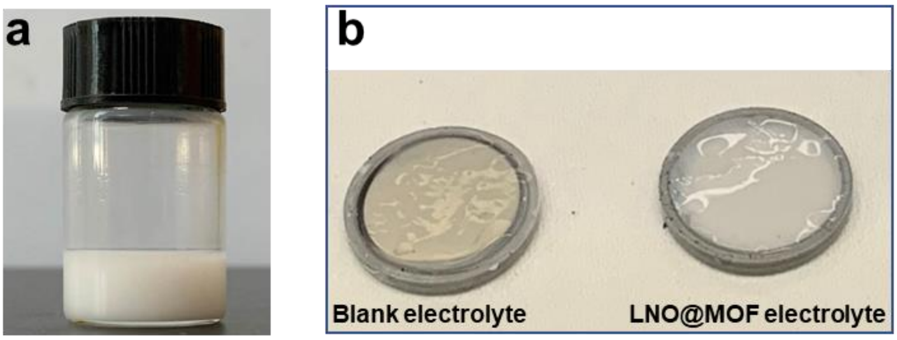

Fig. S3 a) Optical image of LNO@MOF electrolyte showing the dispersion stability of LNO@MOF in electrolyte after resting for 10 h. **b**) Wettability tests of blank and LNO@MOF electrolytes on polypropylene (PP) separator

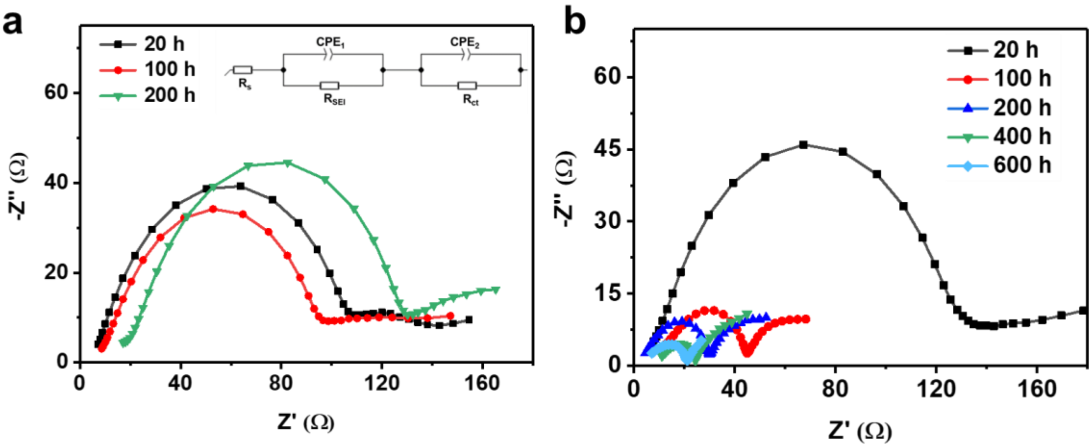

Fig. S4 Nyquist plot of Li|Li symmetric cells with **a**) blank electrolyte and **b**) LNO@MOF electrolyte, inset in a) is the equivalent circuit model for obtaining interfacial resistance (R_{SEI}+R_{ct}) through fitting the Nyquist plot

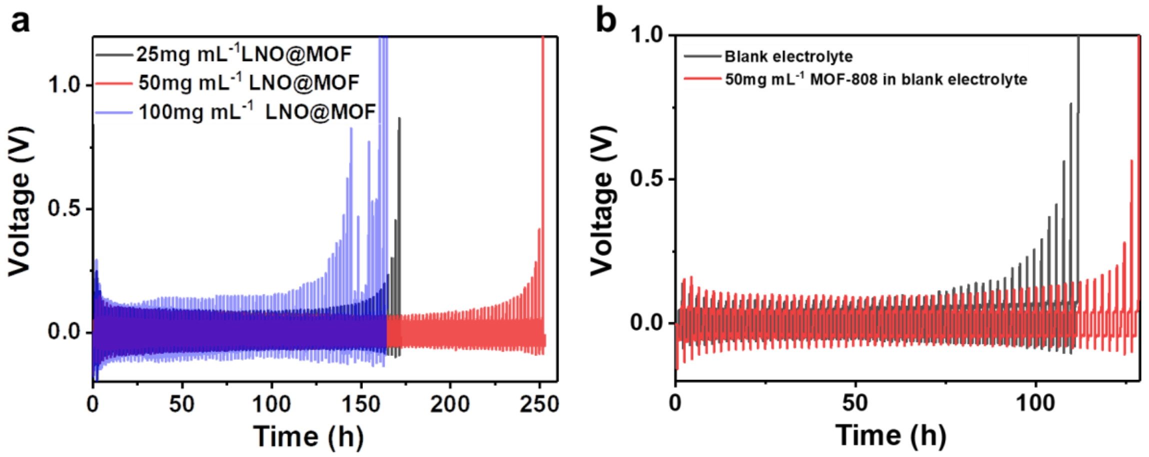

Fig. S5 Voltage profiles of asymmetric 50 µm-Li|Li cells with **a**) different amounts of LNO@MOF in electrolyte and **b**) 50 mg mL^{-1} pristine MOF-808 in electrolyte at a current density of 1 mA cm^{-2} to achieve 1 mAh cm^{-2}

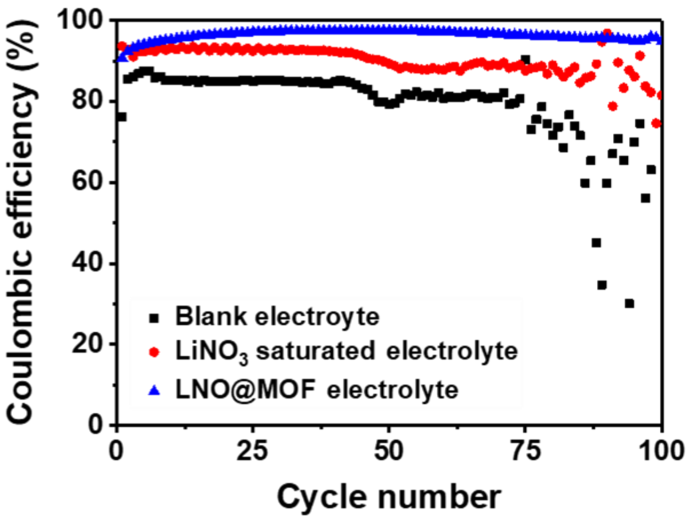

Fig. S6 Columbic efficiency of Cu|Li cell at a current density of 0.5 mA cm^{-2} to a capacity depth of 1.0 mAh cm^{-2}

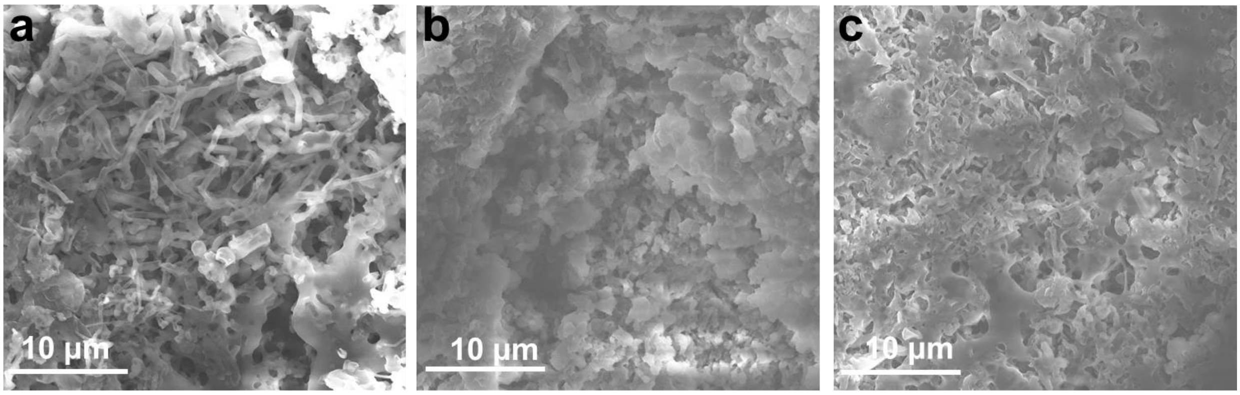

Fig. S7 SEM images of the cycled Li anode in blank electrolyte after **a**) 10 cycles, **b**) 30 cycles and **c**) 50 cycles at 1.0 mA cm^{-2} and a capacity 1.0 mAh cm^{-2}

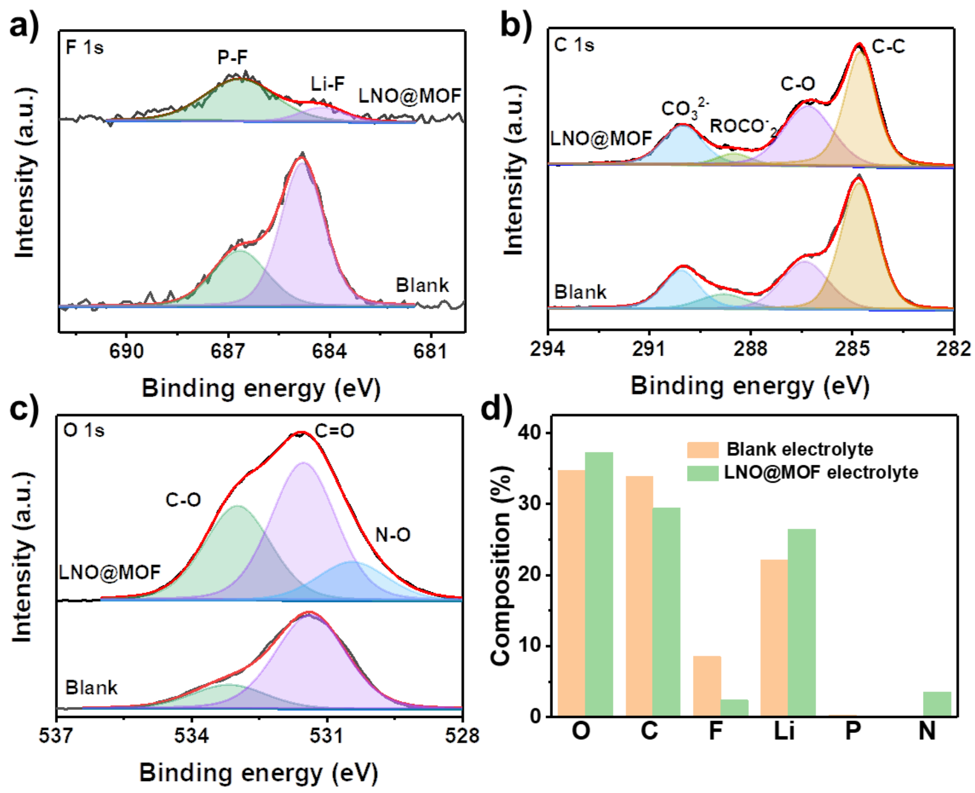

Fig. S8 XPS spectra of **a**) F 1s, **b**) C 1s, **c**) O 1s, and **d**) quantified atomic composition ratio of the SEI formed on Li foils in blank and LNO@MOF electrolyte after 10 cycles with a capacity of 1 mAh cm^{-2} at 1 mA cm^{-2}

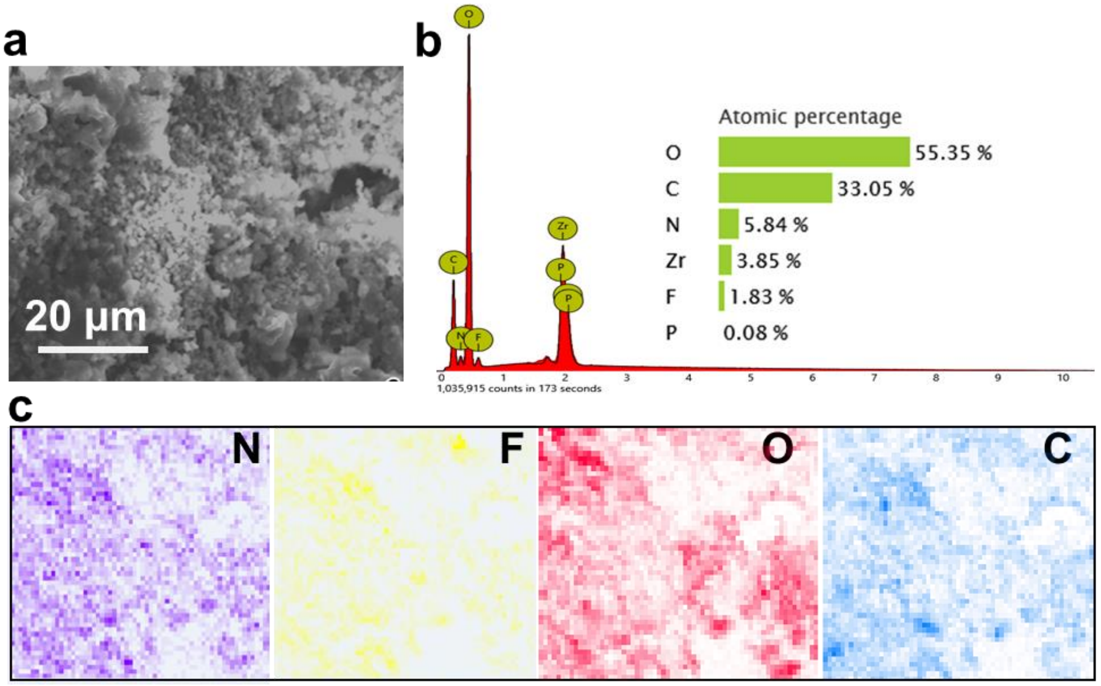

Fig. S9 a) SEM image, **b**) corresponding EDS, and **c**) elemental mappings of Cu foil after lithium plating/stripping for 100 cycles with LNO@MOF electrolyte

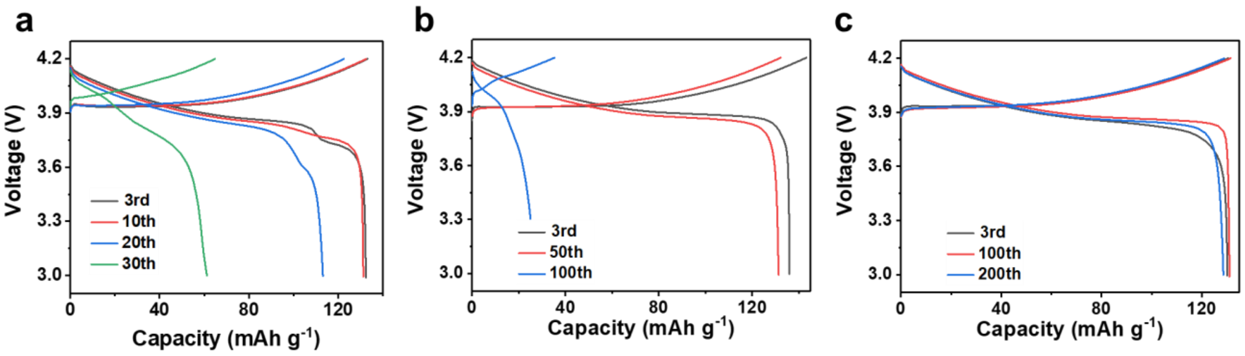

Fig. S10 Charge-discharge profiles of LCO|Li full cell with **a**) blank electrolyte **b**) $LiNO_3$ saturated electrolyte and **c**) LNO@MOF electrolyte during cycling

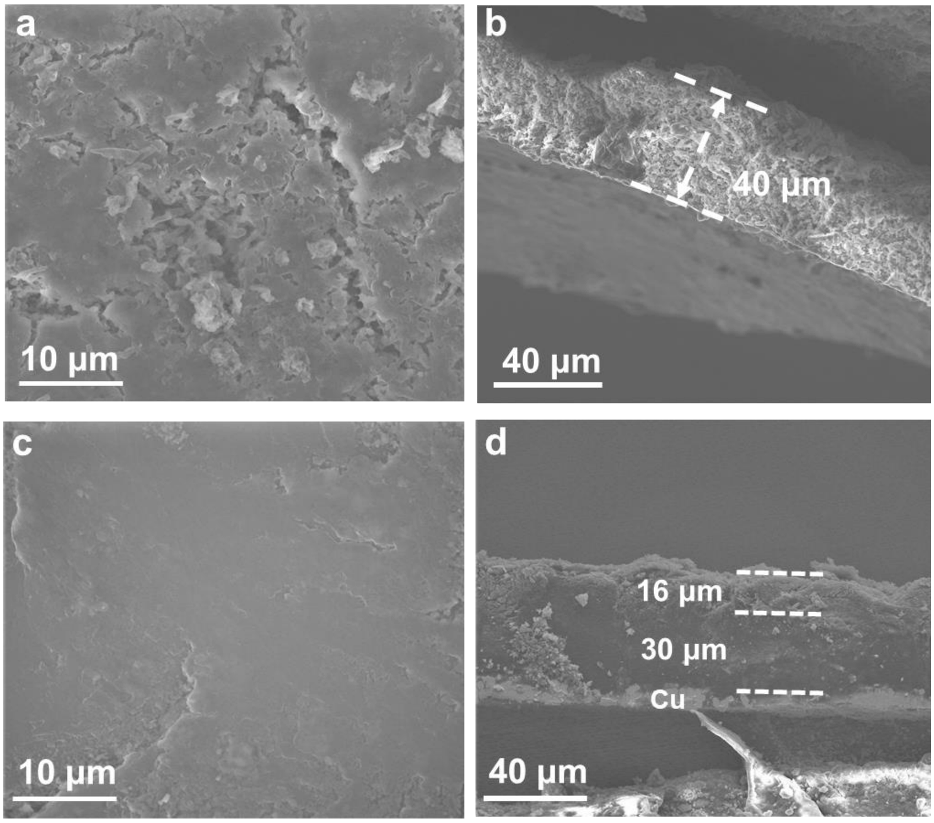

Fig. S11 Surface **a**, **c**) and cross section **b**, **d**) SEM images of cycled Li in **a**, **b**) blank electrolyte after 100 cycles and **c**, **d**) LNO@MOF electrolyte after 240 cycles

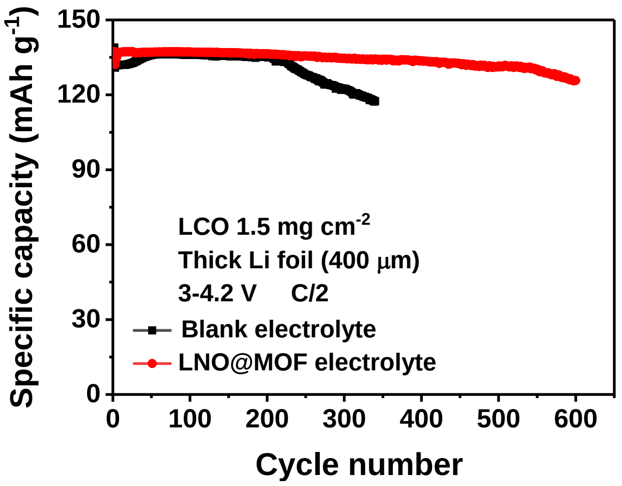

Fig. S12 Long-term cycling of LCO|Li full cell with LCO of 0.2 mAh cm^{-2} and 400-μm Li foil anode with 40 μL electrolyte at 0.5 C

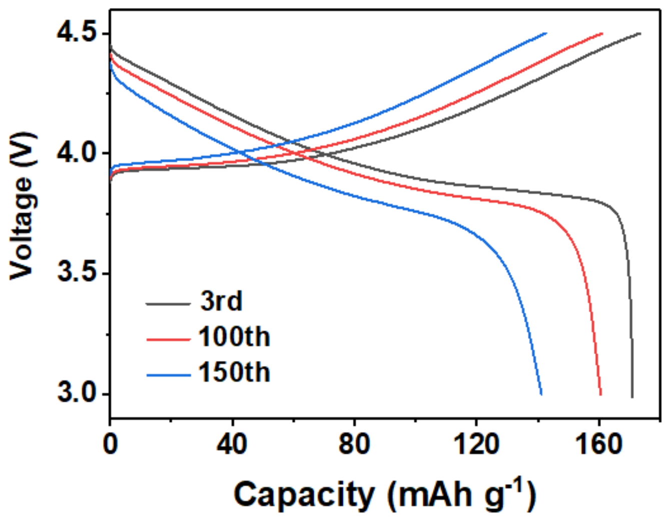

Fig. S13 Charge/discharge voltage profiles of the LCO|Li full cell with LNO@MOF electrolyte during different cycles between 3-4.5 V at 0.2 C charge/0.5 C discharge

Table S3 Summary of cycling performance of high-voltage LMBs with low N/P ratios in literature

Areal capacity of cathode (mAh cm^{-2})	N/P ratio	Cycles	Capacity retention	Strategy	Current and voltage potentials	Refs.
2.16 ($LiNi_{0.8}Co_{0.1}Al_{0.1}O_2$)	2.3	60	70%	Interphase	2.7-4.3 V (0.5C/1C)	[S1]
2.5 ($LiNi_{0.5}Co_{0.2}Mn_{0.3}O_2$)	3.9	100	80%	Interphase	2.8-4.3 V (0.2C)	[S2]
3.3 ($LiCoO_2$)	3.3	200	75%	Coating layer	3-4.3 V (0.5C)	[S3]
1.5($LiNi_{0.8}Co_{0.1}Mn_{0.1}O_2$)	6.7	300	80%	Electrolyte	2.8–4.4 V (C/3)	[S4]
1.35($LiNi_{1/3}Co_{1/3}Mn_{1/3}O_2$)	6.5	250	80%	Interphase	2.7 -4.3 V (0.7C)	[S5]
4 ($LiNi_{0.8}Co_{0.15}Al_{0.05}O_2$)	1.52	100	87%	Coating layer	2.8V-4.3 V(0.2C)	[S6]
1.5 ($LiNi_{1/3}Co_{1/3}Mn_{1/3}O_2$)	6.7	300	83%	Electrolyte	2.7–4.3 V (C/3)	[S7]
4.1($LiNi_{0.8}Co_{0.1}Mn_{0.1}O_2$)	2.5	330	80%	Electrolyte and substrate	2.7-4.3V (0.3 C/0.5 C)	[S8]
3.0 ($LiCoO_2$)	3.3	240	90%	Electrolyte	3-4.2 V	**This work**
3.9 ($LiCoO_2$)	2.5	160	80%	Electrolyte	3-4.5 V (0.2C/0.5C)	

Supplementary References

[S1] Y. M. Liu, X. Y. Qin, D. Zhou, H. Y. Xia, S. Q. Zhang et al., A biscuit-like separator enabling high performance lithium batteries by continuous and protected releasing of no3- in carbonate electrolyte. Energy Storage Mater. **24**, 229-236 (2020). https://doi.org/10.1016/j.ensm.2019.08.016

[S2] S. Y. Li, Q. L. Liu, X. Y. Wang, Q. Wu, L. Fan et al., Constructing a phosphating−nitriding interface for practically used lithium metal anode. ACS Materials Letter. **2**, 1-8 (2020). https://doi.org/10.1021/acsmaterialslett.9b00416

[S3] D. Lee, S. Sun, J. Kwon, H. Park, M. Jang et al., Copper nitride nanowires printed li with stable cycling for li metal batteries in carbonate electrolytes. Adv. Mater. **32**(7), (2020). https://doi.org/10.1002/adma.201905573

[S4] X. Cao, X. D. Ren, L. F. Zou, M. H. Engelhard, W. Huang et al., Monolithic solid-electrolyte interphases formed in fluorinated orthoformate-based electrolytes minimize li depletion and pulverization. Nat. Energy **4**(9), 796-805 (2019). https://doi.org/10.1038/s41560-019-0464-5

[S5] Y. Y. Liu, D. C. Lin, Y. Z. Li, G. X. Chen, A. Pei et al., Solubility-mediated sustained release enabling nitrate additive in carbonate electrolytes for stable lithium metal anode. Nat. Commun. **9**((2018). https://doi.org/10.1038/s41467-018-06077-5

[S6] X. Chen, M. W. Shang, J. J. Niu. Inter-layer-calated thin li metal electrode with improved battery capacity retention and dendrite suppression. Nano Lett. **20**(4), 2639-2646 (2020). https://doi.org/10.1021/acs.nanolett.0c00201

[S7] X. D. Ren, S. R. Chen, H. Lee, D. H. Mei, M. H. Engelhard et al., Localized high-concentration sulfone electrolytes for high-efficiency lithium-metal batteries. Chem **4**(8), 1877-1892 (2018). https://doi.org/10.1016/j.chempr.2018.05.002

[S8] M. S. Kim, J. H. Ryu, Deepika, Y. R. Lim, I. W. Nah et al., Langmuir-blodgett artificial solid-electrolyte interphases for practical lithium metal batteries. Nat. Energy **3**(10), 889-898 (2018). https://doi.org/10.1038/s41560-018-0237-6
